# Enhancing mineral transportation systems in underground mines: A framework for capacity analysis

**DOI:** 10.1016/j.heliyon.2025.e42708

**Published:** 2025-02-14

**Authors:** Mohammad Javad Rahimdel, Reza Mohammadpour

**Affiliations:** Department of Mining Engineering, Faculty of Engineering, University of Birjand, Birjand, Iran

**Keywords:** Underground mining, Mineral transportation system, Production capacity, FTA, Fuzzy logic

## Abstract

The transportation of materials in mining constitutes more than half of the operating costs. The efficient operation of mineral transportation systems significantly influences the production capacity and cost-effectiveness of mining operations. The occurrence of unavoidable failures within these systems presents a substantial production risk. Consequently, investigating the primary causes of failures and assessing the resulting production losses due to operational interruptions have become critical issues in mining engineering. However, there have been limited studies on the capacity analysis of mining operations through the failure analysis of mineral transportation systems. In response to this challenge, this paper introduces an enhanced framework for conducting capacity analysis of mineral production in underground mining operations using Fault Tree Analysis (FTA) and Event Tree (ET), which are well-established techniques in risk management. Given the lack of sufficient data, FTA can be applied to problems within a fuzzy environment. The proposed framework integrates FTA and ET within a fuzzy logic context to comprehensively evaluate system performance. By conducting a case study on a mineral transportation risk assessment at the Qaleh-Zari underground copper mine, the effectiveness of this approach is demonstrated. In this study, all potential scenarios that could lead to mineral transportation failures were identified, and the risks associated with each scenario, as well as the overall risk to the system, were assessed to evaluate potential production losses. The results of this study provide valuable guidance to mine managers, directors, and contractors, enabling them to optimize operational procedures, enhance equipment safety, and effectively mitigate risks associated with mineral production during underground mining operations.

## 1 Introduction

Currently, underground mining contributes approximately 12–17 % of global metal ore production, while surface mining accounts for over 80 %. Additionally, underground mining generates significantly less waste compared to open-pit mining [1,2]. The primary production operations in underground hard rock mining include drilling, blasting, loading, and transportation. The mineral transportation system is a crucial component of underground mines, linking the surface with the below-ground levels of the mining operation [3]. This system is utilized to transport workers and equipment, as well as to bring ore to the surface. The haulage system comprises three primary subsystems: the surface subsystem, the subsystems within the vertical or inclined shaft, and the subsystem situated at the underground levels. Any failure in these subsystems disrupts mineral transportation operations, ultimately resulting in increased overall costs of mineral extraction, decreased transportation quality, and compromised safety for operators. The production capacity of transportation systems is significantly impacted by failure detection and analysis. Additionally, maintenance costs for mining equipment are estimated to account for 30 %–50 % of overall mine operating costs [4]. Consequently, any failure in this equipment leads to an increase in the operating costs of the mines and, ultimately, the price of mineral production [5]. Therefore, it is crucial to investigate the severity and frequency of failures, as well as to analyze the production losses of minerals resulting from unexpected equipment and system failures.

The throughput capacity of the transportation system is crucial for managers and engineers in the decision-making process related to both the design and optimization of production processes. Early detection of incipient faults in the mineral transportation system can minimize breakdowns and, consequently, reduce maintenance time. Therefore, it is essential to study the failure behavior, reliability, and risk analysis of mineral transportation to enhance production efficiency.

Various studies on risk analysis in the mining and mineral industries are available in the literature. However, limited research has focused on the risks associated with production capacity during the underground mining process. Most studies have concentrated on health and safety issues. For example, safety risk analysis [6], damage and injury analysis [7], and health risk analysis [8] have been highlighted. Additionally, the analysis of the failure behavior of mining equipment has been addressed in numerous studies, including the reliability analysis of shovels [9], drilling machines [10], mining trucks [11] and railcars in mines [5]. Nowadays, numerous qualitative, quantitative, and semi-quantitative methods have been employed in risk analysis, including benefit analysis, probability tree analysis, fault tree analysis, scenario planning, event tree analysis, and failure mode and effects analysis. Among these techniques, Fault Tree Analysis (FTA) is one of the most recognized symbolic logic analytical methods. As systems become increasingly complex, the demand for comprehensive analytical techniques to identify all potential combinations of failures that could compromise the integrity of the system is growing [12]. The Fault Tree Analysis (FTA) is a valuable technique for analyzing undesirable events. FTA identifies all potential causes of these events and reveals both qualitative and quantitative interdependencies among them [13]. FTA is a cause-and-effect analysis in which an undesired event is considered the top event. The sub-events that contribute to the occurrence of the top event are referred to as basic events. In traditional FTA, the failure probabilities of basic events must be known with certainty, represented as precise numerical values. However, it cannot handle uncertainties, does not accommodate linguistic variables, and fails to integrate human error into the failure logic model [14]. Additionally, estimating a precise failure rate is challenging due to insufficient data for statistical inference and the ambiguous characteristics of the events [15,16]. In the absence of accurate data, the experiences of field experts provide an effective database for estimating the required data (probability of failures). In such situations, linguistic variables are employed to capture human judgments. To address the limitations of conventional FTA, fuzzy set theory has been proposed to replace crisp numbers with fuzzy numbers, thereby improving the estimation of the likelihood of top events.

Fuzzy set theory was developed as a generalization of classical methods. In a crisp set, an element can either fully belong or not belong at all, whereas a fuzzy set extends this concept by allowing for partial membership. Nowadays, fuzzy set theory is frequently applied as an enhancement to FTA in various fields. Bekbaki et al. [17] utilized fuzzy fault tree analysis for safety risk assessment in chemical process industries. In their study, two types of failure possibility distributions, comprising five and six scales, were employed to estimate the probability of hazardous events occurring. In the aforementioned study, minimal cut sets were prioritized based on importance measures, and process failures were identified as the critical causes of the top event. Cheliyan and Bhattacharyya [18] utilized fuzzy fault tree analysis to investigate the failure of oil and gas leakage in a subsea production system. In this study, expert elicitation and fuzzy set theory were employed to calculate the failure probabilities of the basic events that led to leakage in the offshore pipeline system. The minimal cut sets, which represent the necessary and sufficient conditions for the occurrence of the top event, were identified. Subsequently, several importance measure techniques were applied to determine the critical minimal cut sets, thereby identifying the weakest minimal cut sets that could potentially cause leakage in the subsea production system. Yazdi et al. [16] employed fuzzy fault tree analysis in conjunction with multi-attribute decision-making methods within a fuzzy environment. In their study, fuzzy AHP was utilized to calculate their study the weights assigned to experts. The probabilities of each basic event that could lead to fire and explosion in the process industry were determined by aggregating the weights of the experts. Subsequently, the fuzzy TOPSIS method was applied to prioritize these basic events. The study successfully identified critical basic events and compared them with conventional importance measures. Krechowicz [19] introduced a geotechnical risk management model for horizontal directional drilling operations. In this study, a hybrid approach combining fuzzy fault and event tree analysis was implemented to enhance precision by obtaining crisp probability values for the basic events. The findings of this study revealed combinations of risk remediation strategies that effectively reduce the risk associated with top events. Akhtar and Kirmani [20] employed fuzzy fault tree analysis to evaluate the reliability of wind energy systems. In their study, the failure probabilities of all basic events were determined based on expert opinions. Subsequently, the precise contribution of each basic event to system failure was identified using the fuzzy risk index. Zhao et al. [21] combined fuzzy fault tree analysis with Bayesian networks for navigational risk assessment. In their research, a fault tree model was developed, and the probabilities of basic events were calculated using fuzzy sets. The conditional probabilities of related events were computed, and the probability distribution of adverse consequences was estimated by mapping the fault tree to a Bayesian network. The results of this study highlighted the significance of various factors that contribute to the risk of navigational accidents.

Many risk analyses related to underground mining operations have been conducted within a fuzzy environment to address the ambiguity and uncertainty associated with qualitative factors. Mahdevari et al. [22] assessed the risks associated with human health and safety in underground coal mines. In their study, a methodology based on fuzzy TOPSIS was employed to mitigate the uncertainty of qualitative data. The results identified the most significant risks that have the highest negative impact on the health and safety of personnel working in underground coal mines in Iran. Bakhtavar and Yousefi [23] employed multi-goal fuzzy cognitive mapping and multi-criteria decision-making methods to evaluate the risks associated with workplace accidents in underground collieries. In their study, safety, operational downtime, operational costs, and capital costs were considered as the primary objectives of the analysis. A sensitivity analysis was conducted to prioritize safety by considering various potential weights for these goals. Iphar and Cukurluoz [24] applied the decision matrix method based on the expert knowledge and engineering judgment expressed in linguistic terms, for safety risk analysis in mechanized underground coal production in Turkey. In their paper, risk evaluation was performed using fuzzy logic to address the uncertainties inherent in traditional decision matrix risk assessment methods. Zhang et al. [25] investigated the risk of coal and gas outbursts during underground coal mining operations. In this study, the weights of the outburst risk indicators were determined using the fuzzy analytic hierarchy process, followed by the variable weight theory. The researchers employed sensitivity analysis to address the uncertainties in outburst risk assessment and to evaluate how changes in index parameters affect the variable weights. Wang et al. [26] analyzed the risk factors in mine metro tunneling through expert consultations, utilizing fuzzy set theory, the Analytic Hierarchy Process, and Bayesian Networks (BN). To manage uncertainty in the risk assessment, Pythagorean fuzzy numbers were applied in conjunction with a Bayesian network for expert evaluations.

Various risk analysis studies concerning the failure and reliability of mining vehicles and systems are included in the literature. Table 1 summarizes the existing research on evaluating the risks associated with equipment and systems in the mining and mineral industries. Li et al. [27] proposed an intelligent fault diagnosis method that integrates fault tree analysis with fuzzy neural networks. In this study, fault tree analysis was utilized to identify the causes of failures, followed by the application of neural network inference for fault diagnosis. The proposed methodology was utilized for fault diagnosis of the hydraulic excavator. Özfırat et al. [28] employed the failure mode and effects analysis (FMEA) approach to examine the risks associated with mineral transfer operations involving truck and LHD (Load-Haul-Dump) machines. In this study, the risks encountered during the transfer of minerals from the LHD to underground trucks were identified and ranked based on their probability, severity, and detectability. The findings of the reviewed study indicated that failures in gallery slopes, insufficient gallery widths, and dust accumulation were identified as high-priority risks. Petrovic et al. (2020) developed an algorithm that integrates fuzzy theory with statistical methods for assessing the risks associated with mining machinery. This algorithm prioritizes component failures based on their severity, frequency, and detectability. According to the findings of the aforementioned study, the risk level of the mobile crushing machine was classified as high, indicating the need for an appropriate maintenance policy. Rahimdel and Ghodrati [5] identified the critical failure modes of mine rolling stock by integrating multi-attribute decision-making methods with failure mode and effects analysis. In their study, the analytical hierarchy process was applied within a fuzzy environment to determine the relative importance of each failure mode. The fuzzy inference process was employed to rank the machine's failure modes based on the severity, occurrence, and detection probability of each mode. The results of the aforementioned study indicated that hollow wear of the wheelsets was the primary type of damage to the wheels, leading to the deterioration of the wheel-rail interface. Jin et al. [29] integrated fault tree analysis and Bayesian networks to assess the reliability of the electro-mechanical braking system in mine hoists. In this study, a simulation approach was also conducted to determine the optimal model of the system.

**Table 1:** Summary of reviewed literature on risk assessment of mining equipment.

Reference	The main goal of the implemented approach	Risk analysis methodology implemented	Problem investigated	Case study
[30]	Identification of the related fatal incidents in US mining	Preliminary hazard assessment	The most severe and frequent hazards with high risk level were identified.	Loader and dozer
[31]	Operational risk management during the tunneling	FTA and AHP	All possible risks of mechanized tunneling were identified and categorized.	Tunnel boring machine, TBM
[32]	Critically analysis and maintenance management	FMEA and FMECA	A risk management strategy was proposed for establishing an optimal maintenance program.	Excavator
[3]	Safety risk analysis in underground mining operation	FTA	Root causes of accidents were Identified.	Mine hoist
[28]	Risk evaluation of ore transfer operations to underground trucks	FMEA	Potential failures were identified, and corrective actions and precautions were proposed for all associated risks.	Load-haul-dump, LHD
[33]	Operational risk and financial benefit analysis	Dynamic cash flow model	The financial benefits were modeled, and their total operational losses were assessed.	Ventilation system
[34]	Failure risk analysis of mining machinery	Fuzzy logic	The severity of failures was assessed.	Crushing equipment
[24]	Safety risk evaluation considering the likelihood and severity of the hazard		Risky situations and operations associated with mechanized underground coal mining were evaluated.	Underground coal mining equipment
[5]	Prioritization of the failure modes in mining rolling stocks	FMEA and AHP	Undesired states were identified and discussed.	Railcar
[35]	Reliability evaluation for safe operation of gas tunnel	Bayesian network	The failure probability of a ventilation system was estimated.	Ventilation system
[36]	Proposing a maintenance strategy that takes into account the safety factors relevant to the mining industry.	Fuzzy logic	An approach was developed for the maintenance management of mining machinery, utilizing risk-based maintenance analysis.	Maintenance operation of mining machineries
[37]	Reliability-based risk assessment for a mining auxiliary machine	FMEA	The overall risk of a loader was estimated through the risk priority numbers.	Loader
Current study	Production losses associate with transportation system	Fuzzy logic and FTA	The production risk is evaluated by analyzing the failure probability of equipment	Mineral transportation system

Reviewing the aforementioned papers reveals that, although numerous studies in the literature address the identification of the primary causes of failures and the most hazardous failure modes in the mining industry, there is a lack of research focused on production risk and, consequently, the throughput capacity of mining equipment and systems. Capacity analysis provides essential information for production engineers regarding optimal operation scheduling and bottleneck detection. Production scheduling is predominantly influenced by the production quantities determined during the planning phase. Another important outcome of capacity analysis is the emphasis on subsystems and their improvement, which can enhance the overall production system. This enhancement can be achieved through sensitivity analysis and bottleneck detection.

This paper aims to propose an approach for the production risk and, accordingly the capacity analysis of the mineral transportation system in an underground mine in which an improved production capacity analysis of aggregating fuzzy opinions is proposed. The proposed methodology was demonstrated by applying the fault tree analysis and event tree modeling under the fuzzy environment. This approach provides intuitive, clear, and logical results, enabling both qualitative and quantitative assessments. The unwanted failures of the mineral transportation system were identified for the main components related to the drift tunnels, shaft, and hoist. Moreover, the sensitivity analysis through the importance measures techniques is applied to determine the individual contribution of the basic events that lead to the occurrence probability of the top event.

The results of this study offer a modeling approach to prevent production losses during mineral hauling, as well as a theoretical foundation for identifying the factors that critically impact the availability of mineral transportation systems in underground mines.

The paper is organized as follows: Section 2 introduces the research methodology. The proposed approach is applied to the production capacity analysis of the Qaleh-Zari copper mine in Iran in Section 3. Section 4 evaluates the outputs of the study.

## 2 Research methodology

**Fig. 1:**
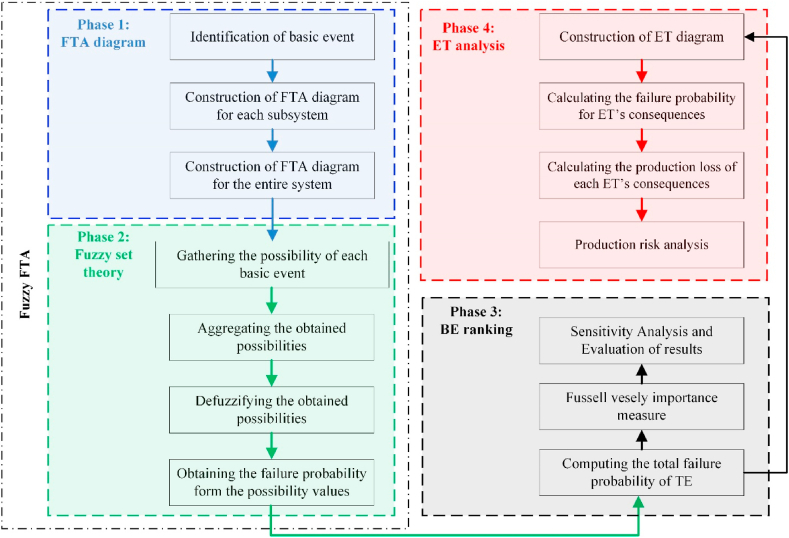
Flowchart used for the production risk analysis.

This section presents a methodology based on Fault Tree Analysis (FTA) and Event Tree Analysis (ETA) within a fuzzy environment for analyzing production loss and capacity in mineral transportation. The proposed methodology is illustrated as a framework in Fig. 1 (phases 1 to 3). In Phase 1, the primary causes of failure are identified, and the Fault Tree (FT) diagram is constructed under the guidance of experts. In Phase 2, the likelihood of basic event failures is determined using expert judgment in a fuzzy environment, after which the failure probabilities are calculated from these likelihood values. In Phase 3, the Event Tree (ET) of the mine's haulage system is developed to outline the sequences of failure events. The production losses, and consequently the overall production losses of the haulage system, are calculated and analyzed during this phase. The framework described above is elaborated upon in the remainder of this section.

### 2.1 Phase 1: Constructing the fault tree

**Fig. 2:**
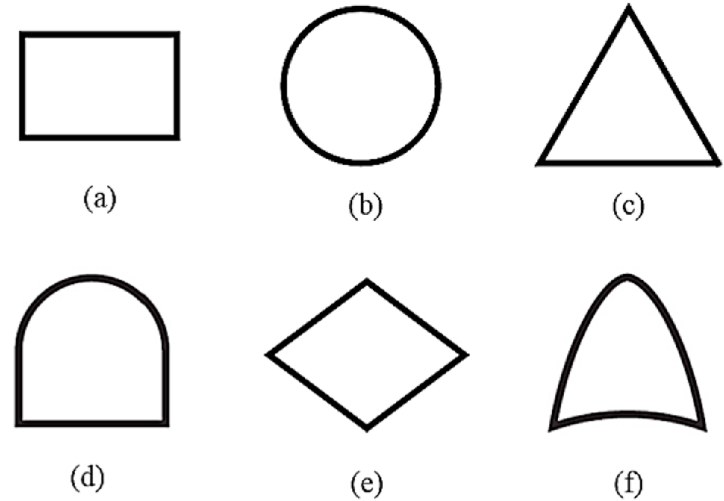
Symbols used in the fault tree [44].

Fault Tree Analysis (FTA) is a well-established method for conducting cause-and-effect analyses in failure analysis [38]. FTA traces a system failure back to the failures at lower levels. In this analysis, a system is represented as a logical structure where the sequence of basic events (causes) leads to top events [[39], [40], [41]]. According to Smith [42], FTA can identify the risks associated with complex systems, assess the consequences of errors, and estimate failure probabilities. The construction of the fault tree begins with the undesirable event, referred to as the top event (TE), and progresses down to the most basic failure modes that lead to the occurrence of the TE, known as basic events (BE) [43]. In this method, to determine the occurrence probability of the TE, the probabilities of the outputs of the logic gates are estimated. To achieve this, a logic diagram is constructed using the events and logic gates. Fig. 2 illustrates the symbols used to construct fault trees. In Fig. 2, the simples (a)–(f), represent an event, a basic event, a transfer symbol, an AND gate, an undeveloped event, and an OR gate, respectively.

In the FTA method, the probability of occurrence for the TE is determined by the probabilities of the Basic Events (BEs) associated with the OR and AND gates. The OR gate represents a scenario in which the output event occurs if at least one of the input events occurs. Conversely, the output of an AND gate is true only if all of its input events are true [15]. The probability of the TE, utilizing the OR and AND gates, is calculated using the following equation [45,46]:

(1) $$P_{TE}=\left\{ \begin{array}{c} 1-\prod_{i=1}^{m}\left(1-P_{i}\right)\;for\;OR\;gate\\ \prod_{i=1}^{m}P_{i}\;for\;AND\;gate \end{array}\right.$$

Where, $P_{TE}$ is the occurrence probability of the top event associated with the OR and AND gates, $P_{i}$ is the probability of each *i*th basic event, and *m* is the number of basic events.

### 2.2 Phase 2: Calculation of the failure probabilities for BE

The failure probability of each basic event is calculated in the following steps.

Step 1. Gathering the possibility of the basic events

After creating the FT diagram, the probabilities of BE are determined using the failure database. Due to the lack of failure probability data for the mineral transportation equipment and systems, the failure probabilities were estimated through expert judgment. Since the failure rates of the components are imprecise or vague, the probability of the top event cannot be accurately calculated using classical probability theory. To address this limitation, fuzzy set-based techniques are typically employed [47,48].

The fuzzy set theory, introduced by Zadeh [49], addresses the vagueness and imprecision inherent in human judgment during decision-making. This theory generalizes classical set theory by allowing for degrees of membership. Unlike conventional sets, which permit only full or non-membership of an element, fuzzy set theory accommodates a spectrum of membership values. In fuzzy set theory, partial membership is also considered, along with full and no membership. The linguistic terms such as very low, medium, and high are used to reflect the human thinking style in the decision-making process. The data, collected from experts in terms of linguistic variables, are represented using mathematical concepts derived from fuzzy set theory. This approach addresses the imprecision and uncertainty inherent in human judgment.

A fuzzy set *A* is an ordered pair, $A=\{\left(x,\mu_{A}\left(x\right)\right)|x\in X,\mu_{A}\left(x\right):X\to \left[0,1\right]\}$, where *X* is a collection of objects, and $\mu_{A}\left(x\right)$ is the membership function which indicates the membership grade of the elements of *X* in the fuzzy set *A*. It is important to note that each element *x* in *X* takes on absolute numerical values between 0 and 1 to represent a degree of membership. Triangular and trapezoidal membership functions are the most commonly used types of membership functions in fuzzy set theory. In this paper, the trapezoidal membership functions are used. Considering *x*_1_, *x*_2_, *x*_3_, and *x*_4_ as trapezoid's base points, the trapezoidal fuzzy membership function (*μ*_*A*_(*x*)) can be expressed using Eq. (2) [50]:

(2) $$\mu_{A}\left(x\right)=\left\{ \begin{array}{c} \begin{array}{l} \frac{x-x_{1}}{x_{2}-x_{1}}\;x_{1}\leq x\leq x_{2}\\ 1\;x_{2}\leq x\leq x_{3} \end{array}\\ \begin{array}{l} \frac{x-x_{4}}{x_{3}-x_{4}}\;x_{3}\leq x\leq x_{4}\\ 0\;otherwise \end{array} \end{array}\right.$$

In this paper, the Similarity Aggregation Method (SAM), developed by Hsu and Chen [51], is utilized to aggregate distinct linguistic opinions from experts. The SAM is the most effective method for assessing a non-homogeneous group of experts. This approach has been widely utilized to evaluate risk in different industries, such as the marine industry [52,53], medicine [54], manufacturing [55], aviation [56], mining industry [56], and agriculture [57].

Step 2. Aggregating the obtained possibilities

Assume that a total of *M* experts, denoted as *E*_*r*_ (*r* = 1, 2, …, *M*), express their opinions on the likelihood of failure for each basic event using a set of linguistic variables. In the initial step of the SAM, the linguistic terms provided by the experts are converted into corresponding fuzzy numbers. Subsequently, the aggregated fuzzy numbers are derived following the procedure [58].

1) *Calculating the agreement degree*

The degree of agreement is calculated to quantify the similarity between the opinions of various experts. The similarity function (S) is employed to compute the level of similarity between two fuzzy values. Consider $\tilde{R_{u}}=\left(a_{1},a_{2},a_{3},a_{4}\right)$ and $\tilde{R_{v}}=\left(b_{1},b_{2},b_{3},b_{4}\right)$ as the opinion of experts $E_{u}$ and $E_{v}$ in the form of two standard trapezoidal fuzzy numbers. The degree of similarity between the opinions $E_{u}$ and $E_{v}$ is calculated from Eq. (3):

(3) $$S\left(\tilde{R_{u}},\tilde{R_{v}}\right)=1-\frac{1}{4}\sum_{i=1}^{4}\left|a_{i}-a_{i}\right|$$

Where, $S\left(\tilde{R_{u}},\tilde{R_{v}}\right)\epsilon \left[0,1\right]$ is the degree of similarity between the trapezoidal membership numbers $\tilde{R_{u}}$ and $\tilde{R_{v}}$. The greater value $S\left(\tilde{R_{u}},\tilde{R_{v}}\right)$ means there is the best similarity between two experts concerning the fuzzy numbers.

2) *Computing the weighted agreement degree of the experts*

In the third step, the weighted agreement degree for each expert $E_{u}$ is calculated as Eq. (4);

(4) $$WA\left(E_{u}\right)=\frac{\sum_{\begin{array}{c} v=1\\ v\neq u \end{array}}^{M}W\left(E_{v}\right).S\left(\tilde{R_{u}},\tilde{R_{v}}\right)}{\sum_{\begin{array}{c} v=1\\ v\neq u \end{array}}^{M}W\left(E_{v}\right)}$$

Where, $WA\left(E_{u}\right)$ is the weighted agreement degree, $W\left(E_{u}\right)$ is the weight of the expert $E_{u}$. The importance assigned to each expert's evaluation is referred to as the expert's degree of importance. The calculation of an expert's degree of importance is based on factors such as their experience, knowledge, and professional position. For each expert, the importance degree ($W\left(E_{u}\right)$) is calculated considering the weighing score of expert factors ($WA\left(E_{u}\right)$) as Eq. (5):

(5) $$W\left(E_{u}\right)=\frac{WA\left(E_{u}\right)}{\sum_{a=1}^{M}WA\left(E_{u}\right)}$$

3) *Calculating the relative agreement degrees of the experts*

The relative agreement degree of the experts (*RA*($E_{u}$)) is determined as Eq. (6):

(6) $$RA\left(E_{u}\right)=\frac{WA\left(E_{u}\right)}{\sum_{u=1}^{M}WA\left(E_{u}\right)}$$

4) *Computing the consensus coefficient degree of the experts*

The consensus coefficient degree of the experts $E_{u}\left(u=1,\ldots ,M\right)$ is computed by using Eq. (7):

(7) $$CC\left(E_{u}\right)=\beta .W\left(E_{u}\right)+\left(1-\beta \right).RA\left(E_{u}\right)$$

Where $CC\left(E_{u}\right)$ is the consensus coefficient degree of the experts $E_{u}$ and β is a relaxation factor between 0 and 1. Regarding Hsu and Chen [51], the relaxation factor is crucial for balancing the relative degrees of agreement and the importance degree (*W*) assigned by an expert. The *β* factor determines the significance of *W*(*E*_*a*_) in relation to *RA*(*E*_*a*_). This factor is assigned by decision-makers based on their preferences. This factor serves as an appropriate means to evaluate the relative value of the expert's opinion. When *β* equals zero, expert weights are disregarded, resulting in the use of a homogeneous group of experts. Conversely, when *β* equals one, the consensus among the experts reflects the importance weights. The consensus coefficient for each expert offers a valuable measure for assessing the relative significance of each opinion [[58], [59], [60]]. However, it is important to note that expert judgment is fundamentally based on uncertainty and ambiguity; therefore, conducting an uncertainty analysis is crucial in this context [[61], [62], [63]].

5) *Calculating the aggregated result of the judgment*

To obtain the aggregated result of the expert's judgment ($\tilde{R}$), the overall fuzzy number is calculated as Eq. (8):

(8) $$\tilde{R}=\sum_{u=1}^{M}CC\left(R_{u}\right).{\tilde{E}}_{u}=CC\left(R_{1}\right).R_{1}+CC\left(R_{2}\right).{\tilde{R}}_{2}+\ldots +CC\left(R_{M}\right).{\tilde{R}}_{M}$$

Step 3: Defuzzifing the aggregated expert judgment

To convert the aggregated results of the experts' judgments into a fuzzy possibility score, a defuzzification process is employed. Various algorithms can be utilized for this purpose, including the Center of Area (COA) [64], the Bisector of Area (BOA), the Mean of Maximum (MOM) method [65], the Smallest of Maximum (SOM), and the Largest of Maximum (LOM) methods [66]. In this paper, the center of the area method is used. In the COA method, the defuzzified output ($X^{*}$) for a trapezoidal fuzzy set number ($a_{1},a_{2},a_{3},a_{4}$) is obtained from Eq. (9):

(9) $$X^{*}=\frac{\int_{a_{1}}^{a_{2}}\frac{x-a_{1}}{a_{2}-a_{1}}xdx+\int_{a_{2}}^{a_{3}}xdx+\int_{a_{3}}^{a_{4}}\frac{a_{4}-x}{a_{4}-a_{3}}xdx}{\int_{a_{1}}^{a_{2}}\frac{x-a_{1}}{a_{2}-a_{1}}dx+\int_{a_{2}}^{a_{3}}xdx+\int_{a_{3}}^{a_{4}}\frac{a_{4}-x}{a_{4}-a_{3}}dx}=\frac{1}{3}\frac{{\left(a_{4}+a_{3}\right)}^{2}-a_{4}a_{3}-{\left(a_{1}+a_{2}\right)}^{2}+a_{1}a_{2}}{a_{3}+a_{4}-a_{1}-a_{2}}$$

Step 4: Calculating the fuzzy failure probabilities

The possibility values of each BE are converted into the probability values. Onisawa [67] proposed the Eq.s (10) and (11) to transform fuzzy failure possibilities into fuzzy failure probabilities.

(10) $$\mathrm{Pr}=\left\{ \begin{array}{c} \frac{1}{10^{k}},FPS\neq 0\\ 0,FPS=0 \end{array}\right.$$

(11) $$k={\left[\left(\frac{1-FPS}{FPS}\right)\right]}^{\frac{1}{3}}\times 2.301$$

where, Pr is the failure probability, *FPS* is the crisp failure possibility, and *k* is a constant that represents the safety criterion based on the lower bound of the error rate and the error rates of a routine. It is important to note that, in the Onisawa formula, the error rate is assumed to be 10^−2^–10^−3^ and the lower bound of the error rate is 5 × 10^−5^ [67].

### 2.3 Phase 3: Computing the overall failure probability of TE and BE ranking

The probability of TE occurrence is calculated based on the probabilities of BEs associated with OR and AND gates, as outlined in Eq. (1). In the FTA procedure, a Minimal Cut Set (MCS) is defined as the smallest combination of BEs that leads to the occurrence of a TE [68]. The FT can contain numerous MCSs; however, it is essential to identify those that include the most critical components. The mathematical representation of the MSCs in an FT can be expressed as Eq. (12) [69,70]:

(12) $$TE={MCS}_{1}+{MCS}_{2}+\ldots +{MCS}_{n}={⋃}_{i=1}^{n}{MCS}_{i}$$

where, *n* is the number of MCS.

The probability of TE occurrence considering the MCS is calculated as Eq. (13) [69,70]:

(13) $$P\left(TE\right)=P\left({MCS}_{1}\cup {MCS}_{2}\cup \ldots \cup {MCS}_{n}\right)=P\left({MCS}_{1}\right)+P\left({MCS}_{2}\right)+\ldots +P\left({MCS}_{n}\right)-P\left({MCS}_{1}\cap {MCS}_{2}\right)+\left(P\left({MCS}_{1}\cap {MCS}_{2}\right)+P\left({MCS}_{1}\cap {MCS}_{3}\right)+\ldots \right)+{\left(-1\right)}^{n-1}P\left({MCS}_{1}\cap {MCS}_{2}\cap \ldots \cap {MCS}_{n}\right)$$

where, *P*(*MCS*_*i*_) is the occurrence probability of *MCS*_*i*_ and *n* is the number of MSC.

Identifying the roles of components and their significance in the occurrence of the top event is the most critical aspect of FTA during risk assessment. This approach identifies the subsystems or items that require greater attention to enhance the system's performance. To achieve this, sensitivity analyses utilizing importance measure techniques are conducted to evaluate the individual contributions of each BE that leads to the TE. In this paper, the Fussell-Vesely (FV) importance measure is employed to prioritize BEs based on their contributions to the occurrence of the TE. The FV importance measure is the probability that at least one minimal cut set containing event *i* has failed at time *t*, given that the system has also failed at that same time. This can be obtain in FTA as Eq. (14) [[71], [72], [73]]:

(14) $${IM}_{i}^{FV}=\frac{\sum_{j=1}^{n}P_{j}^{i}}{P_{T}}$$

Where, ${IM}_{i}^{FV}$ is the importance measure of *i*th basic event, $P_{j}^{i}$ denotes the probability that *j*th MCS which contain BE *i* is failed, *n* is the number of MCS, and $P_{T}$ is the occurrence probability of the TE.

The importance analysis for MCS identifies the most critical combinations that lead to the occurrence of TE. The importance measure of *i*th MCS (${IM}_{{MCS}_{i}}^{FV}$) is calculated utilizing Eq. (15) [[74], [75], [76]]:

(15) $${IM}_{{MCS}_{i}}^{FV}=\frac{P\left({MSC}_{i}\right)}{P_{T}}$$

Where, $P\left({MSC}_{i}\right)$ is the failure probability of *i*th MCS.

After identifying the critical BEs using the importance measure tool, sensitivity analysis is employed to understand the behavior of uncertainties within a system. This analysis provides a quantitative assessment that helps identify the weakest relationships between input events and the appropriate design to be integrated into a specific system [77,78]. Sensitivity analysis assists decision-makers in pinpointing significant sources of variability and uncertainty during the risk assessment process [[77], [79], [80]]. In this paper, to address uncertainty in FTA concerning the varying weights assigned to different experts, sensitivity analysis is applied using a variation of the relaxation factor (*β*), as shown in Eq. (7). This approach investigates how changes in expert judgment influence the final prioritization of basic events.

### 2.4 Phase 4. Constructing the event tree and computing the production risk

The Event Tree (ET) is a horizontal structure designed to model the initiating event of a failure. There are two types of ET used in probabilistic risk analysis: functional and systemic. In the functional ET, the loss of mitigating functions is treated as events to understand the scenario of events at an abstract level, ultimately leading to an initiating event. The systemic ET outlines the scenarios that result in a failure by depicting the overall system's response to an initiating event [68,81].

In this phase, sequences of failure events are delineated using the systemic ET structure. This approach models all possible scenarios leading to failure by defining all potential failure sequences associated with a specific initiating event. To construct the ET diagram, the initial event is positioned on the left side, while all related subsequent events are arranged on the right side in sequential order. The initial event is connected to the first subsequent event with a lateral line. The occurrence of each event in the FT depends on the preceding events in the sequence. The success states of each event are represented at the top of the lateral line, while the failure states are indicated at the bottom.

After the construction of the ET, all potential scenarios that could lead to system failure are identified. This approach allows for the assessment of the risk associated with each scenario, as well as the overall risk to the system.

## 3 Numerical risk analysis for production loss assessment in underground mine

In this section, a numerical risk analysis was conducted using FTA and ETA within a fuzzy environment to assess the production losses in the mineral transportation system at the Qaleh-Zari copper mine.

### 3.1 Qaleh-Zari Copper Mine

Qaleh-Zari underground copper mine is located 180 km south of Birjand city in the South Khorasan province of Iran and on the northern the Lut Desert. The mining site covers an area of approximately 6 square kilometers, with mineralization widths ranging from 0.5 to 7 m. Qaleh-Zari is the only underground mine in Iran that employs shrinkage-stopping methods.

The shrinkage-stopping method is primarily used for non-layered and steep ore deposits. In this method, crushed ore is transported to the loading area by gravitational force. The extraction process involves drilling and blasting. Shrinkage-stopping is a vertical and upward mining method. The foundation of this method is to retain approximately two-thirds of the blasted ore within the mining stope, which not only facilitates access to the roof for drilling and blasting operations but also provides support for the hanging and foot walls.

**Fig. 3:**
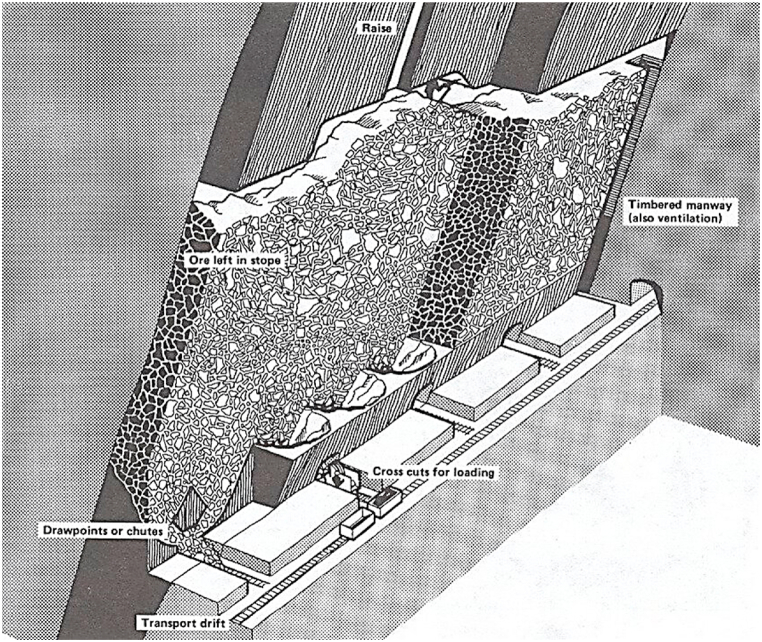
Shrinkage stoping in a large vertical ore body [83].

Fig. 3 illustrates the layout of shrinkage stoping in a large vertical body. As previously mentioned, the extraction direction in this method is upward, meaning that the working face progresses in an upward direction. The ore is extracted through horizontal cuts from the bottom to the top, with crushed ore accumulating in the mined areas. In this method, the mineral fills the mining stope, thereby supporting the walls. Simultaneously, the remaining broken ore within the stope serves as a platform for drilling and blasting operations in the upper sections. As the volume of extracted ore increases, some of the broken mineral must be removed from the stope at designated draw points; otherwise, the blasted minerals may obstruct the stope. Most of the extracted ore is stored in stopes. When the distance between the mining floor of the crushed stones and the floor of the stope becomes minimal, it becomes impossible to continue mining operations. Consequently, some of the broken ore is discharged, while the remainder is stored in the stope until the end of the operation [82].

The mining operation at the Qaleh-Zari Mine began in 1975 and is still ongoing. The development process for accessing the ore deposit at Qaleh-Zari Mine involves drilling vertical wells, constructing drift tunnels, and ultimately creating man-ways and ore pass raises. The extracted ores are transported to the surface via vertical shafts, including one inclined shaft (the main shaft). Currently, there are seven in-operation shafts at the Qaleh-Zari Mine. The specifications for these shafts are provided in Table 2.

**Table 2:** Specifications of in-operation shafts of Qaleh-Zari Copper Mine.

Shaft no.	Depth (m)	Production (ton/month)
1	190	2437.7
2	210	2039.4
3	130	969.1
4	145	979.8
5	170	965.7
6	215	851.5
7	90	188.7

**Fig. 4:**
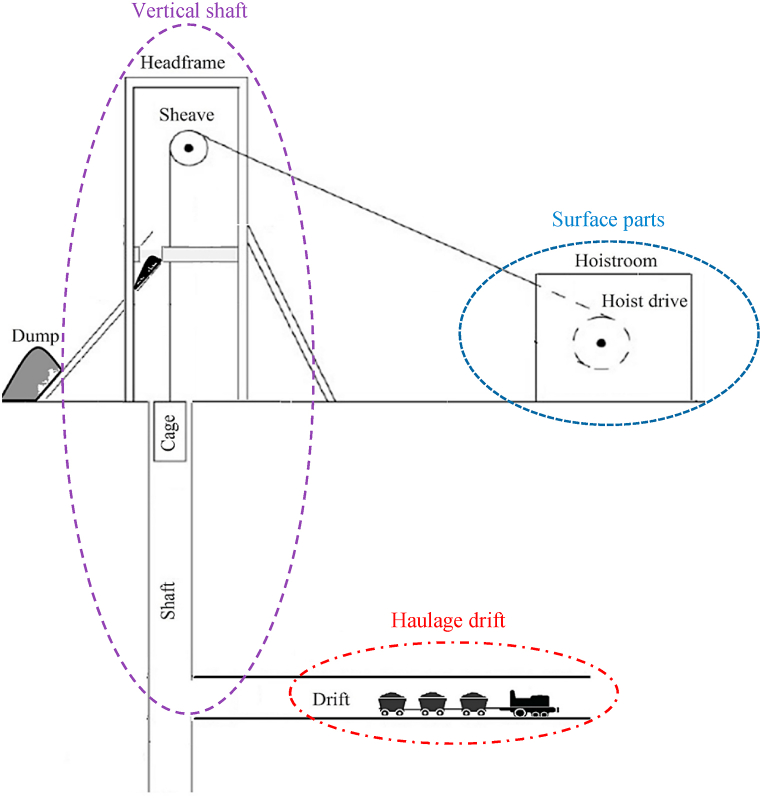
The layout of the mineral transportation system in underground mines (Adapted from [3]).

The broken ore is discharged from the chutes located at the bottom of the stope into crosscuts, where it is loaded into wagons using pneumatic loaders. The extracted ore is then transported to the main shaft through the transport drift and finally lifted to the surface using a single cage. Fig. 4 illustrates the layout of the mineral transportation system at the Qaleh-Zari Copper Mine. This system comprises numerous subsystems, components, and items. As shown in Fig. 4, the primary components of the mineral transportation system can be categorized into three main groups: surface equipment (including winches, drums, and other mechanical and electrical devices), equipment in the vertical shaft (including cages and rails), and equipment in drift tunnels and crosscuts (including bunkers and material transfer facilities).

This mine is the biggest underground copper mine in Iran. However, due to the passage of over 40 years since the commencement of mining operations, along with aging equipment and machinery, the productivity of mineral production has significantly declined. Therefore, it is essential to analyze production risks and quantify the losses in mineral production resulting from equipment failures. This approach will not only identify critical equipment and machinery but also improve the production process and effectively mitigate risks associated with mineral production.

### 3.2 Problem statement

In underground mining operations, the haulage system is a crucial component of the transportation network, responsible for transferring minerals, equipment, and miners from different levels of the mine to the surface. A disruption in the mineral transportation system can result in decreased production, increased operating costs, and potentially lead to a complete stoppage of the mineral transport. Consequently, the primary objective of this study is to investigate the risks associated with transportation operations at the Qaleh-Zari mine using FTA and ETA within a fuzzy environment.

The primary question addressed in this paper is summarized as follows:

- What are the primary causes of failure in the mine's mineral transportation system?
- How likely is each basic event to fail?
- How much is the failure probability for each shaft, as well as the total failure probability of the mineral transportation system?
- Which factors contribute most significantly to the failure of mineral transportation?
- How much production capacity is lost in the mineral transportation system at the Qaleh-Zari copper mine?

In this paper, the stoppage of shaft production is determined as the TE. Subsequently, a FT diagram is constructed based on the systematic arrangement of BEs that lead to the interruption of mineral transportation. To achieve this, all potential failure causes are identified for each shaft. The FT diagram for each shaft is developed under the guidance of underground mining experts, after which the FT for the entire mine production network is created. The likelihood of failure for the Basic Events is determined using the fuzzy aggregation method, and the failure probabilities are then calculated from these likelihood values. In this approach, the failure probability for each shaft and the overall mineral transportation system of the mine are obtained. Following this, the ET for the mine's haulage system is constructed to illustrate the sequences of failure events. In this phase, the production losses for each shaft, and consequently the production losses for the mine's haulage system, are calculated and analyzed.

## 4 Results

**Fig. 5:**
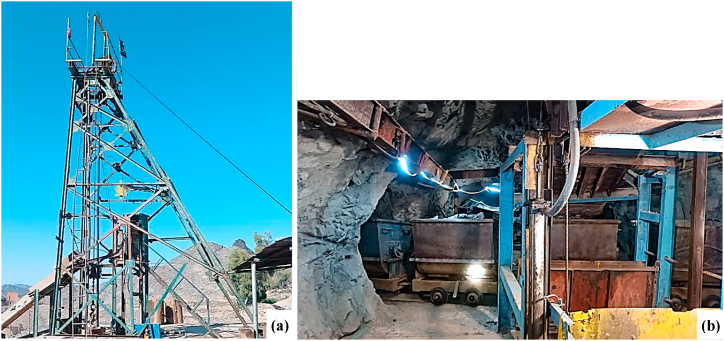
The (a) headframe and (b) drift tunnel of Qaleh-Zari Copper Mine.

**Fig. 6:**
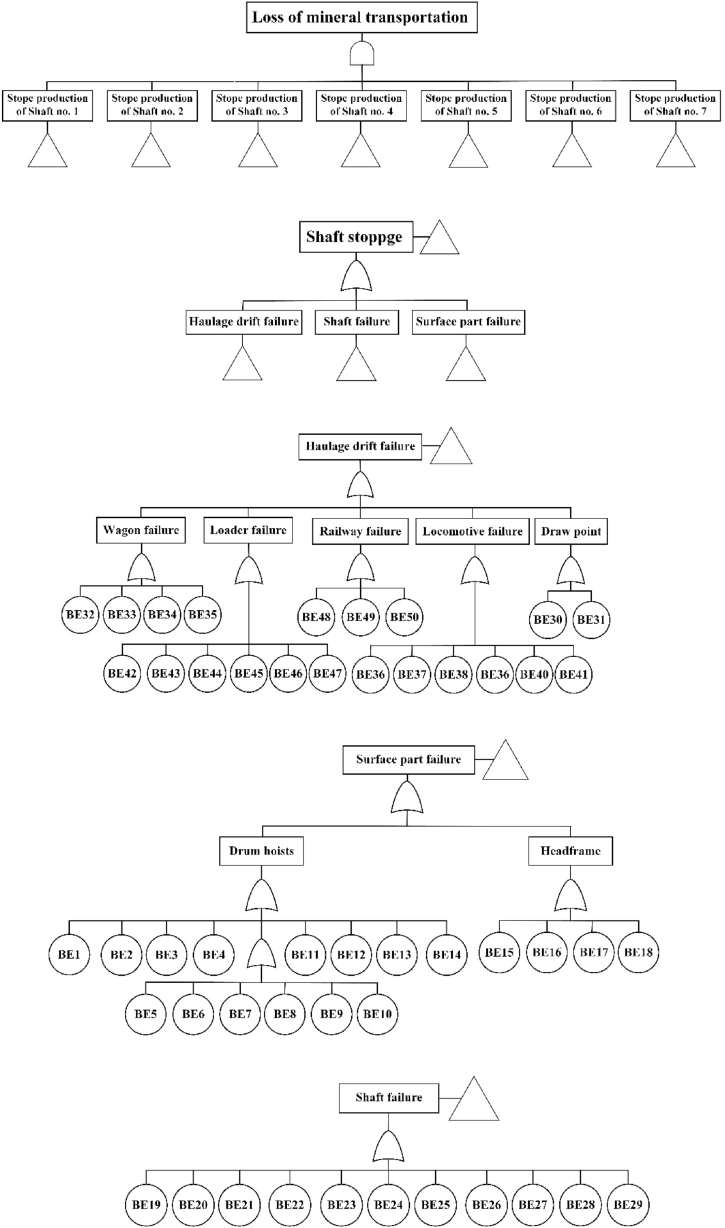
Fault tree diagram.

This section is dedicated to applying the FTA to estimate production losses resulting from the mineral transportation system. The first step involves constructing the FT diagram to identify the Top Event (TE) and Basic Events (BEs). Losses in mineral transportation from each shaft may arise from failures related to equipment in the haulage drift, vertical shaft (hoisting system), or surface operations. Fig. 5 illustrates the headframe and haulage drift of a shaft at the Qaleh-Zari Copper Mine. Data necessary for this analysis was collected from underground mine experts with extensive experience in the operation and maintenance of various components of the mineral transportation system at the Qaleh-Zari Copper Mine. A group of seven experts (four for each shaft) was selected, including shaft supervisors, drum drivers, loader and locomotive operators, and workers from the mine under study, to identify all potential BEs that could lead to interruptions in mineral transportation. Following a thorough examination of failure reports and consultations with experts, all possible BEs that directly contribute to failures in the mineral transportation system are listed in Table 3. Additionally, the FT diagram constructed to analyze mineral production loss is presented in Fig. 6.

**Table 3:** BE for mineral transportation system.

Part	No.	FTA definition	Potential failures
Surface part (hoisting system)	1	BE1	Brake pad failure (Winch)
	2	BE2	Sheave brake failure (Winch)
	3	BE3	Rupture of disc brake spring (Winch)
	4	BE4	Wear and tear of brake shoes (Winch)
	5	BE5	Clutch failure (Winch)
	6	BE6	Hydraulic system failure (Winch)
	7	BE7	Body failure (Winch)
	8	BE8	Brake dust (Winch)
	9	BE9	Hydrostator drum malfunction (Winch)
	10	BE10	Electrical connectors (Winch)
	11	BE11	Electromotor failure (Winch)
	12	BE12	Gearbox failure (Winch)
	13	BE13	Inverter malfunction (Winch)
	14	BE14	Shaft door (Headframe)
	15	BE15	Head frame body (Headframe)
	16	BE16	Dumping system (Headframe)
	17	BE17	Motor failure (shaft door)
	18	BE18	Drive sheave failure (Headframe)
Vertical shaft	19	BE19	Shaft walls
	20	BE20	Wire rope (damage and abrasion)
	21	BE21	Dumping bucket hook
	22	BE22	Dumping bucket body
	23	BE23	Support frame (failure and decay)
	24	BE24	Connectors failure
	25	BE25	Sheave failure
	26	BE26	Safety guide of dumping bucket (failure and decay)
	27	BE27	Shaft door
	28	BE28	Dumping bucket spring
	29	BE29	Warning bell
Haulage drift	30	BE30	Lever (Draw point)
	31	BE31	Body corrosion (Draw point)
	32	BE32	Bearing failure (Wagon)
	33	BE33	Wagon connections (Wagon)
	34	BE34	Wagon frame (Body and chassis)
	35	BE35	Wheels (Wagon)
	36	BE36	Body and chassis (Locomotive)
	37	BE37	Gearbox failure (Locomotive)
	38	BE38	Motor failure (Locomotive)
	39	BE39	Battery (Locomotive)
	40	BE40	Wheels (Locomotive)
	41	BE41	Connections (Locomotive)
	42	BE42	Body and frame (Loader)
	43	BE43	Main motor (Loader)
	44	BE44	Boom (Loader)
	45	BE45	Wheels (Loader)
	46	BE46	Pneumatic hoses and connectors (Loader)
	47	BE47	Erosion of wire rope and chain (Loader)
	48	BE48	Braces (Railway)
	49	BE49	Sleeper (Railway)
	50	BE50	Track decay and fracture (Railway)

The expert judgment is utilized to determine the probability of the BE. It is important to note that the experts participating in the study vary in age, educational background, professional roles, and experience. Consequently, the weighting assessment procedure outlined in the methodology section is employed to obtain the significance of each expert. The weighting scores for the experts are presented in Table 4.

**Table 4:** Classification of experts and their corresponding scores.

Parameter	Classification	Score
Professional position (P1)	Manager	10
	Academic professor	8
	Engineer	6
	Technician	4
	Worker	2
Work Experience (P1)	>30	10
	21–30	8
	16–20	6
	11–15	4
	<5	2
Education level (P3)	Ph.D.	10
	Master	8
	Bachelor	6
	Diploma	4
	School level	2
Age (P4)	>50	8
	41–50	6
	31–40	4
	<30	2

The degree of importance (weight) assigned to the experts involved in the research for all studied shafts is calculated and presented in Table 5.

**Table 5:** Weighting score of experts.

Shaft no.	Expert no.	Weighting score
1	E1	0.211
	E2	0.263
	E3	0.263
	E4	0.263
2	E1	0.307
	E2	0.210
	E3	0.253
	E4	0.230
3	E1	0.184
	E2	0.210
	E3	0.264
	E4	0.342
4	E1	0.257
	E2	0.314
	E3	0.257
	E4	0.172
5	E1	0.294
	E2	0.235
	E3	0.264
	E4	0.207
6	E1	0.229
	E2	0.235
	E3	0.323
	E4	0.213
7	E1	0.250
	E2	0.272
	E3	0.250
	E4	0.228

In this section, a systematic risk analysis has been conducted to predict the production of mining shafts. Following expert assessments, an aggregation method was employed to determine the probabilities of failure. The linguistic expressions provided by mine experts were converted into equivalent fuzzy numbers. Table 6 presents the trapezoidal fuzzy numbers corresponding to the relevant linguistic terms [58,84]. In this approach, the similarity function for all basic events of shaft no. 1 was calculated. Subsequently, the values for weighted agreement (WA), relative agreement (RA), and consensus coefficient (CC) were computed. In this study, the parameter β is set to 0.5 [85,86]. The next step involved aggregating the expert judgments using Eq. (8). Following this, the defuzzification process was carried out to convert fuzzy numbers into crisp values using Eq. (9). The defuzzified failure probabilities for all BEs were calculated, and the fuzzy probability rates (FPT) were derived from the fuzzy possibility rates. An example is provided in Table 7, which details the calculation process for the fuzzy failure probability (FFP) of the first basic event (BE1). A similar procedure was employed to obtain the FFP for the other basic events.

**Table 6:** Linguistic terms and their corresponding trapezoidal fuzzy numbers.

Linguistic term	Fuzzy number
Very Low (VL)	(0,0,0.1,0.2)
Low (L)	(0,0.1,0.2,0.3)
Medium Low (ML)	(0.2,0.3,0.4,0.5)
Medium (M)	(0.4,0.5,0.5,0.6)
Medium-High (MH)	(0.5,0.6,0.7,0.8)
High (H)	(0.7,0.8,0.8,0.9)
Very High (VH)	(0.8,0.9,1,1)

**Table 7:**
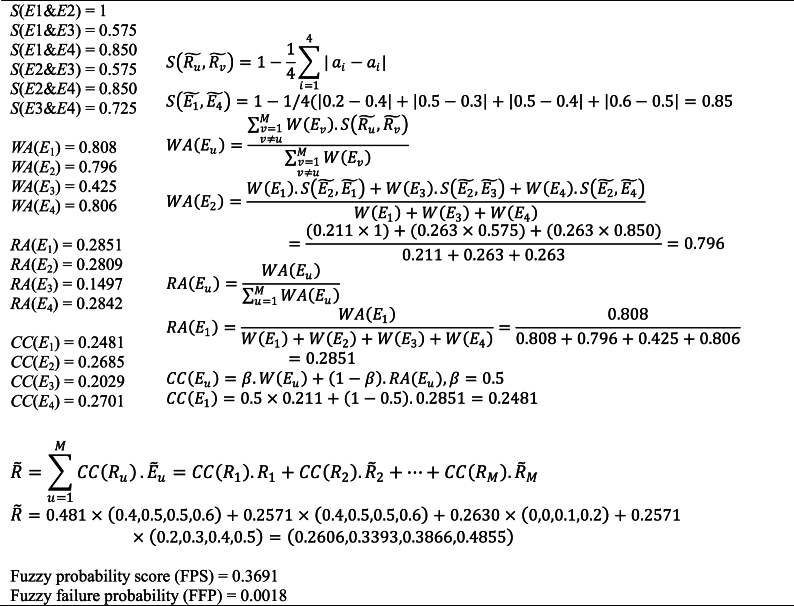
Detailed aggregation and fuzzy failure probability calculation process for BE1.

According to the FT diagram, the probability of occurrence for the top event (loss of mineral transportation) has been calculated for all shafts and is presented in Table 8. To assess the significance of BEs and their impact on the occurrence of the TE, the FV importance measure was computed. Table 9 displays the MCSs and the FV importance rankings of MCSs for all shafts. This approach allows for the identification of basic events that have the greatest influence on the shaft and mineral transportation system.

**Table 8:** Occurrence probability of top events for mine shafts.

Shaft no.	1	2	3	4	5	6	7
Occurrence probability of TE (fuzzy probability score)	0.1391	0.2265	0.2511	0.2632	0.0712	0.1174	0.2052

**Table 9:** MCSs and FV importance rank for each shaft.

MCS	Shaft number						
	1	2	3	4	5	6	7
BE1	19	11	12	38	16	43	6
BE2	9	14	3	47	8	31	19
BE3	37	40	33	42	44	50	44
BE4	34	33	18	33	49	33	34
BE5	44	38	36	13	50	16	46
BE6	30	23	41	43	23	42	48
BE7	49	49	45	48	40	7	49
BE8	20	37	22	45	11	44	30
BE9	40	44	23	49	26	19	36
BE10	32	13	40	34	47	39	33
BE11	39	45	50	19	45	48	5
BE12	13	48	49	16	24	36	20
BE13	15	19	44	32	41	41	38
BE14	36	24	21	36	4	25	21
BE15	26	25	28	46	46	40	37
BE16	41	28	29	29	39	30	26
BE17	17	20	20	23	33	46	50
BE18	21	42	35	12	21	38	15
BE19	45	29	37	41	37	26	45
BE20	35	5	48	30	48	21	25
BE21	28	34	47	21	31	9	11
BE22	8	39	26	7	28	18	2
BE23	31	26	38	39	10	32	18
BE24	24	17	39	26	30	23	9
BE25	25	18	46	20	27	28	22
BE26	22	15	27	24	25	27	35
BE27	10	43	17	25	3	15	17
BE28	18	21	30	35	34	34	29
BE29	14	36	7	10	6	13	24
BE30	11	4	32	3	29	6	41
BE31	12	2	24	11	5	2	12
BE32	42	47	5	8	7	45	40
BE33	3	8	8	2	38	1	1
BE34	2	6	2	17	2	12	10
BE35	5	3	1	5	14	5	3
BE36	47	50	34	50	32	49	42
BE37	7	35	9	18	20	24	13
BE38	43	30	25	27	42	17	4
BE39	27	9	15	15	22	4	14
BE40	6	12	13	6	18	35	8
BE41	38	32	42	28	36	47	43
BE42	48	46	43	37	1	37	39
BE43	23	27	11	44	19	11	32
BE44	46	41	14	40	9	8	27
BE45	50	7	19	31	35	29	31
BE46	1	31	31	1	12	20	16
BE47	4	16	4	4	15	10	7
BE48	16	10	10	9	17	22	47
BE49	29	1	6	14	13	3	23
BE50	33	22	16	22	43	14	28

**Fig. 7:**
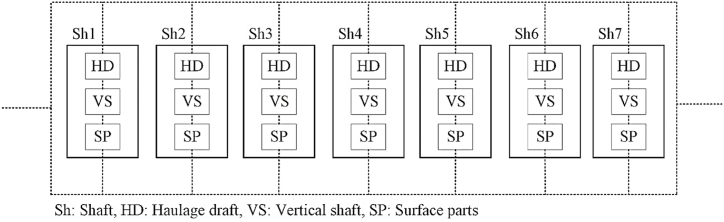
Block diagram of shafts.

**Fig. 8:**
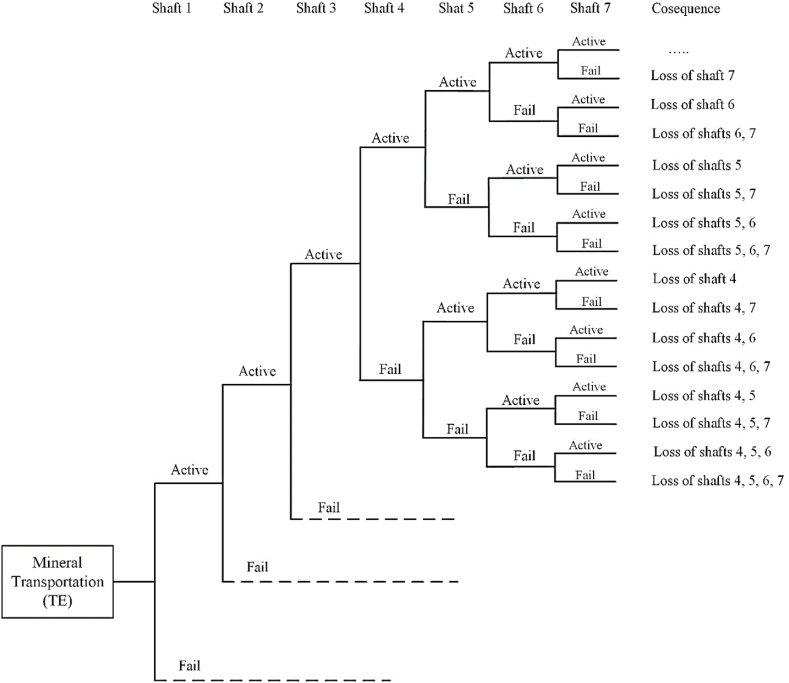
Event tree based on all possible scenarios of shafts.

After calculating the failure probability of TE, all potential consequences that could disrupt the mineral transportation operations of the mine are identified. Using the block diagram of all in-operation shafts in the mine, represented as a serial-parallel network (Fig. 7), a consequence analysis is conducted to complete the production risk assessment. To achieve this, the ET diagram is constructed, as illustrated in Fig. 8. The ET diagram outlines all possible states of the shafts and their corresponding consequences. The risk assessment of the mine shafts, based on various risk scenarios, is computed and presented in Table 10.

**Table 10:** Risk assessment of mineral transportation according to different risk scenarios.

Scenario no.	The failed shaft no.	Failure probability	Average loss of production (t/h)	Risk assessment (t/h)
1	…	0.00E+00	0	0.00E+00
2	7	6.18E-02	2.288	1.41E-01
3	6	8.55E-02	1.565	1.34E-01
4	6,7	2.21E-02	3.853	8.51E-02
5	5	7.01E-02	2.736	1.92E-01
6	5,7	4.65E-02	5.024	2.34E-01
7	5,6	2.50E-02	4.301	1.08E-01
8	5,6,7	6.47E-03	6.589	4.26E-02
9	4	8.03E-02	1.441	1.16E-01
10	4,7	2.07E-02	3.729	7.73E-02
11	4,6,7	2.87E-02	3.006	8.62E-02
12	4,5	7.40E-03	5.294	3.92E-02
13	4,5,7	2.35E-02	4.177	9.82E-02
14	4,5,6	6.07E-03	6.465	3.92E-02
15	4,5,6,7	8.40E-03	5.742	4.82E-02
16	2	2.17E-03	8.030	1.74E-02
17	2,7	3.18E-02	0.590	1.88E-02
18	2,4	8.22E-03	2.878	2.37E-02
19	2,4,7	1.14E-02	2.155	2.45E-02
20	3	2.94E-03	4.443	1.30E-02
21	3,7	9.32E-03	3.326	3.10E-02
22	3,4	2.41E-03	5.614	1.35E-02
23	3,4,7	3.33E-03	4.891	1.63E-02
24	3,2	8.60E-04	7.179	6.17E-03
25	3,2,7	1.07E-02	2.031	2.17E-02
26	3,2,4	2.76E-03	4.319	1.19E-02
27	3,2,4,7	3.81E-03	3.596	1.37E-02
28	6,4	9.85E-04	5.884	5.79E-03
29	6,2	3.13E-03	4.767	1.49E-02
30	6,2,7	8.07E-04	7.055	5.69E-03
31	6,2,4	1.63E-03	6.332	1.03E-02
32	6,2,4,7	4.21E-04	8.620	3.63E-03
33	6,3	1.57E-02	0.406	6.38E-03
34	6,3,7	4.05E-03	2.694	1.09E-02
35	6,3,4	5.85E-03	1.971	1.15E-02
36	6,3,4,7	1.51E-03	4.259	6.42E-03
37	6,3,2	4.62E-03	3.142	1.45E-02
38	6,3,2,7	1.19E-03	5.430	6.47E-03
39	6,3,2,4	1.72E-03	4.707	8.08E-03
40	6,3,2,4,7	4.43E-04	6.995	3.10E-03
41	5,2	8.54E-03	1.847	1.58E-02
42	5,2,7	2.20E-03	4.135	9.11E-03
43	5,2,4	3.18E-03	3.412	1.08E-02
44	5,2,4,7	8.19E-04	5.700	4.67E-03
45	5,3	6.47E-04	6.871	4.44E-03
46	5,3,7	2.51E-03	6.871	1.72E-02
47	5,3,4	9.32E-04	7.307	6.81E-03
48	5,3,4,7	2.40E-04	8.436	2.03E-03
49	5,3,2,7	2.10E-03	0.996	2.10E-03
50	5,3,2,7	5.42E-04	3.284	1.78E-03
51	5,3,2,4	7.82E-04	2.561	2.00E-03
52	5,3,2,4,7	2.02E-04	4.849	9.78E-04
53	5,6,2	6.18E-04	3.732	2.31E-03
54	5,6,2,7	1.59E-04	6.020	9.59E-04
55	5,6,2,4	2.30E-04	5.297	1.22E-03
56	5,6,2,4,7	5.92E-05	7.585	4.49E-04
57	5,6,3	2.95E-03	2.437	7.19E-03
58	5,6,3,7	2.95E-04	4.725	1.39E-03
59	5,6,3,4	4.25E-04	4.002	1.70E-03
60	5,6,3,4,7	1.10E-04	6.290	6.89E-04
61	6,5,3,2	3.36E-04	5.173	1.74E-03
62	5,6,3,2,7	8.65E-05	7.461	6.46E-04
63	5,6,3,2,4	1.25E-04	6.738	8.41E-04
64	5,6,3,2,4,7	3.22E-05	9.026	2.90E-04
65	1	3.21E-02	1.545	4.96E-02
66	1,7	8.28E-03	3.833	3.17E-02
67	1,4	1.19E-02	3.110	3.71E-02
68	1,4,7	3.08E-03	5.398	1.66E-02
69	1,2	9.43E-03	4.281	4.04E-02
70	1,2,7	2.43E-03	6.569	1.60E-02
71	1,2,4	3.51E-03	5.846	2.05E-02
72	1,2,4,7	9.04E-04	8.134	7.35E-03
73	1,3	1.74E-02	2.986	5.21E-02
74	1,3,7	4.50E-03	5.274	2.37E-02
75	1,3,4	6.48E-03	4.551	2.95E-02
76	1,3,4,7	1.67E-03	6.839	1.14E-02
77	1,3,2	5.12E-03	5.722	2.93E-02
78	1,3,2,7	1.32E-03	8.010	1.06E-02
79	1,2,3,4	1.90E-03	7.287	1.39E-02
80	1,2,3,4,7	4.91E-04	9.575	4.70E-03
81	1,6	4.30E-03	2.135	9.17E-03
82	1,6,7	1.11E-03	4.423	4.90E-03
83	1,6,4	1.60E-03	3.700	5.91E-03
84	1,6,4,7	4.12E-04	5.988	2.47E-03
85	1,6,2	1.26E-03	4.871	6.15E-03
86	1,6,2,7	3.25E-04	7.159	2.33E-03
87	1,6,2,4	4.69E-04	6.436	3.02E-03
88	1,2,4,6,7	1.21E-04	8.724	1.06E-03
89	1,3,6	2.33E-03	3.576	8.35E-03
90	1,3,6,7	6.02E-04	5.864	3.53E-03
91	1,3,4,6	8.68E-04	5.141	4.46E-03
92	1,3,4,6,7	2.24E-04	7.429	1.66E-03
93	1,6,3,2	6.85E-04	6.312	4.33E-03
94	1,6,3,2,7	1.77E-04	8.600	1.52E-03
95	1,6,3,2,4	2.55E-04	7.877	2.01E-03
96	1,6,3,2,4,7	6.57E-05	8.467	5.56E-04
97	1,5	2.45E-03	1.951	4.79E-03
98	1,5,7	6.33E-04	4.239	2.68E-03
99	1,5,4	9.12E-04	3.516	3.21E-03
100	1,5,4,7	2.35E-04	5.804	1.37E-03
101	1,5,2	7.21E-04	4.687	3.38E-03
102	1,5,2,7	2.11E-04	6.975	1.47E-03
103	1,5,2,4	2.68E-04	6.252	1.68E-03
104	1,5,2,4,7	6.91E-05	8.540	5.90E-04
105	1,5,3	1.33E-03	3.392	4.52E-03
106	1,5,3,7	3.44E-04	5.680	1.95E-03
107	1,5,3,4	4.96E-04	4.957	2.46E-03
108	1,5,3,4,7	1.28E-04	7.245	9.26E-04
109	1,5,3,2	3.91E-04	6.128	2.40E-03
110	1,5,3,2,7	1.01E-04	8.416	8.50E-04
111	1,5,3,2,4	1.46E-04	7.693	1.12E-03
112	1,5,3,2,4,7	3.75E-05	9.981	3.75E-04
113	1,5,6	3.28E-04	2.541	8.34E-04
114	1,5,6,7	8.47E-05	4.829	4.09E-04
115	1,5,6,4,	1.22E-04	4.106	5.01E-04
116	1,5,6,4,7	3.15E-05	6.394	2.01E-04
117	1,5,6,2,	9.64E-05	5.277	5.09E-04
118	1,5,6,2,7	2.49E-05	7.565	1.88E-04
119	1,5,6,2,4,	3.58E-05	6.842	2.45E-04
120	1,5,6,2,4,7	9.24E-06	9.130	8.44E-05
121	1,5,6,3	1.78E-04	3.982	7.10E-04
122	1,5,6,3,7	4.60E-05	6.270	2.88E-04
123	1,5,6,3,4	6.63E-05	5.547	3.68E-04
124	1,5,6,3,4,7	1.71E-05	7.835	1.34E-04
125	1,5,6,3,2	5.24E-05	6.718	3.52E-04
126	1,5,6,3,2,7	1.35E-05	9.006	1.22E-04
127	1,5,6,3,2,4	1.95E-05	8.283	1.61E-04
128	1,5,6,3,2,4,7	5.02E-06	10.571	5.31E-05

The remainder of this section is dedicated to conducting a sensitivity analysis to validate and clarify the performance of the applied methodology, as indicated by the results presented in Table 10. To achieve this, the priority importance of BEs of all studied shafts was determined by varying the relaxation factor (*β*). For instance, the criticality rankings, considering different relaxation factors for all BEs of shaft No. 1, are shown in Table 11.

**Table 11:** Importance ranks of all BEs for shaft No. 1 considering different relaxation factor.

Basic event	BE Ranking								
	*β* = 0.1	*β* = 0.2	*β* = 0.3	*β* = 0.4	*β* = 0.5	*β* = 0.6	*β* = 0.7	*β* = 0.8	*β* = 0.9
BE1	18	18	18	19	19	19	19	20	20
BE2	9	9	9	9	9	9	9	9	9
BE3	37	37	37	37	37	38	39	40	41
BE4	34	34	34	34	34	34	34	34	34
BE5	42	44	44	44	44	44	44	44	44
BE6	30	30	30	30	30	30	29	29	29
BE7	49	49	49	49	49	49	49	49	49
BE8	20	20	20	20	20	20	21	21	21
BE9	40	40	40	40	40	40	38	38	38
BE10	32	32	32	32	32	32	32	33	33
BE11	38	38	38	38	39	39	40	41	40
BE12	11	11	13	13	13	13	13	13	13
BE13	15	15	15	15	15	15	15	15	15
BE14	35	36	36	36	36	36	36	36	36
BE15	24	24	24	24	26	27	27	27	28
BE16	41	41	41	41	41	42	42	42	42
BE17	19	19	19	18	17	17	17	17	17
BE18	22	21	21	21	21	21	20	18	18
BE19	45	45	45	45	45	45	45	45	45
BE20	36	35	35	35	35	35	35	35	35
BE21	29	29	28	28	28	28	28	28	27
BE22	8	8	8	8	8	7	7	6	6
BE23	31	31	31	31	31	31	31	31	32
BE24	25	25	25	25	24	24	24	24	24
BE25	26	26	26	26	25	25	25	25	25
BE26	21	22	22	22	22	23	23	23	23
BE27	10	10	10	10	10	10	10	10	10
BE28	17	17	17	17	18	18	18	19	19
BE29	14	14	14	14	14	14	14	14	14
BE30	12	12	11	11	11	11	11	11	11
BE31	13	13	12	12	12	12	12	12	12
BE32	44	43	42	42	42	41	41	39	39
BE33	3	3	3	3	3	3	3	3	3
BE34	2	2	2	2	2	2	2	2	2
BE35	5	5	5	5	5	5	5	5	5
BE36	47	47	47	47	47	47	47	47	47
BE37	7	7	7	7	7	8	8	8	8
BE38	43	42	43	43	43	43	43	43	43
BE39	27	27	27	27	27	26	26	26	26
BE40	6	6	6	6	6	6	6	7	7
BE41	39	39	39	39	38	37	37	37	37
BE42	48	48	48	48	48	48	48	48	48
BE43	23	23	23	23	23	22	22	22	22
BE44	46	46	46	46	46	46	46	46	46
BE45	50	50	50	50	50	50	50	50	50
BE46	1	1	1	1	1	1	1	1	1
BE47	4	4	4	4	4	4	4	4	4
BE48	16	16	16	16	16	16	16	16	16
BE49	28	28	29	29	29	29	30	30	30
BE50	33	33	33	33	33	33	33	32	31

## 5 Discussion

Regarding the analysis of basic events, the winch brake (shafts No. 1, 2, and 3), drive sheave of the headframe (shaft No. 4), shaft door of the headframe (shaft No. 5), winch body (shaft No. 6), and electromotor of the winch (shaft No. 7) are the surface parts with the highest probability of failure. Damage and abrasion of the wire rope (shaft No. 1), dumping bucket body (shafts No. 2 and 4), warning well (shaft no. 3), shaft door (shaft No. 5), dumping bucket hook (shaft No. 6), and connectors (Shaft No. 7) exhibit the highest occurrence of failure among all parts of the vertical shafts. An analysis of the failures of the haulage draft parts shows that the pneumatic hoses of loaders (shafts no. 1 and 4), railway sleeper (shaft No. 2), wheels of wagons (shaft No. 3), body and frame of loaders (shaft No. 5), wagon connections (shaft No. 6 and 7) have the greatest likelihood of failure.

The failure likelihood of the shafts indicates that shaft no. 4, with a probability of 0.2632, has the highest occurrence probability compared to the other shafts. The transportation of minerals from this shaft is significantly impacted by the pneumatic loaders. All pneumatic loaders require compressed air at a specific pressure. Insufficient air pressure can result from issues with the compressed air valves and pipe leaks. This leads to a decrease in the efficiency of the pneumatic loaders and, consequently, an increase in energy consumption. Therefore, it is essential to inspect the compressed air hoses and connectors, as well as to monitor the pressure drop in the compressed air distribution lines. Implementing these recommendations will help enhance the reliability of mineral production and reduce the risk of ore production interruptions caused by disruptions in mineral transportation.

Regarding the importance measure analysis, BE 46 (hoses and connectors of loaders), BE 49 (sleeper of railway tracks), BE 35 (wagon wheel), BE 42 (loader body and frame), BE 31 (body corrosion of draw points), and BE 33 (wagon connections) exhibit the highest significance in the failure occurrence of shafts. These findings indicate that leaks in the compressed air network and the wheelsets of the railcars are the primary causes of equipment failure in haulage drifts, consistent with previous studies. Rahimdel and Zafarzadeh [87] examined the adequacy of the flow rate and permissible pressure of compressed air for air consumers in the Qaleh-Zari mine. The findings of this study revealed that the air pressure within the compressed air network lines of the mine falls below the permissible limit. Consequently, it was recommended to change the diameter of the pipes, increase the airflow pressure, and utilize local compressors near the consumers' locations. Numerous studies have shown that wheelsets are a significant cause of railcar failures. According to Rahimdel and Ghodrati [5], overheated wheels and torsion of the wheelset axle represent the most hazardous failure modes of rolling stock. Another study indicated that the wheelset and brake are the most critical subsystems of rolling stock due to the highest incidence of failures and maintenance costs [88]. These studies underscore the importance of investigating the failure behavior of this equipment to ensure safe and efficient transportation while maintaining vehicle performance at an acceptable operational level.

Table 10 illustrates the potential consequences of mineral transportation loss along with their corresponding occurrence probabilities. As shown in Table 10, scenario no. 3, where shaft no. 4 is the only failed shaft, has the highest occurrence probability for loss of mineral production. Scenario no. 6, which involves the failure of shafts no. 2 and 7, presents the highest risk level at 0.235 t/h. Consequently, any failure of shafts no. 2 and 7 significantly increases the risk of mineral transportation, making it the most critical scenario for the mine's mineral production. Scenario no. 5, where shaft no. 2 is the only failed shaft, has the second highest risk for mineral transportation at 0.192 t/h. The total risk associated with mineral transportation is calculated to be 2.351 t/h.

Regarding the sensitivity analysis, the priority ranking of the BEs for all shafts is highly sensitive to changes in the relaxation factor. On the other hand, ranking of BEs is significantly influenced by the magnitude of the relaxation factor. The sensitivity analysis for Shaft No. 1 reveals that the risk priority ranking of only 18 out of 50 BEs (36 %) is same. However, for 23 of the remaining BEs (46 %), the risk priority ranking differs by only one position when different values of the relaxation factor are applied. The risk priority rankings for 42 %, 28 %, 26 %, 10 %, 32 %, 30 %, and 32 % of all identified BEs for Shafts No. 2 to 7, respectively, remained the same. These results indicate that there are significant differences in the BE rankings concerning varying relaxation factor values. Therefore, this factor plays a significant role in the critical analysis and can be obtained from expert opinions. Notably, all critical BEs (those with the highest priority) maintain the same ranking across different scenarios.

## 6 Conclusions

Mining equipment and machinery are among the most critical components in mining operations and production processes. The failure of such equipment can result in production losses, ultimately leading to an increase in the cost of mineral production. Therefore, predicting production risks is essential for decision-making during both the design and operational phases. To address this risk, this study presents a comprehensive framework for capacity analysis of mineral transportation networks within underground mining operations. By identifying potential faults that could lead to mineral transportation losses, we conducted a quantitative risk analysis using FTA in a fuzzy environment, supplemented by ET analysis. The proposed methodology was applied as a case study in the operational shafts of the Qaleh-Zari copper mine in Iran. According to the results of the study, key components such as the sheave brake of the winch, damage and abrasion of wire ropes, and wagon wheels exhibited the highest failure probabilities in surface equipment, vertical shafts, and haulage drifts, respectively. The highest probability of shaft failure was 0.2632. Furthermore, we quantified the total production loss of the mine at 2.35 tons per hour, with shafts No. 2 and No. 7 identified as having the most significant impact on mineral transportation capacity among the seven shafts studied.

These results indicate that establishing a system for recording failure and repair data is essential. This system will be beneficial for future analyses focused on studying the reliability, availability, and maintainability of the equipment, as well as for proposing appropriate preventive maintenance tasks. These findings provide actionable insights for underground mine managers and directors to identify weaknesses in the mine haulage system, facilitating the development of an efficient and reliable mineral transport system. Additionally, they assist in maintaining mineral production rates at planned levels.

While our proposed framework leverages the strengths of FTA and ET within a fuzzy environment, it is not without limitations. The data collection process for estimating failure occurrences relies on expert judgments, which may introduce inaccuracies and time constraints. Additionally, the model's inability to capture statistical dependencies between basic events may lead to challenging results. Future research should explore additional risk factors to enhance the accuracy of risk predictions and address statistical dependencies to achieve more robust modeling outcomes.

## CRediT authorship contribution statement

**Mohammad Javad Rahimdel:** Writing – review & editing, Writing – original draft, Visualization, Supervision, Software, Methodology, Investigation, Formal analysis, Conceptualization. **Reza Mohammadpour:** Software, Investigation, Formal analysis, Data curation.

## Data availability statement

Data will be made available on request. For requesting data, please write to the corresponding author.

## Funding

The current study did not receive any specific grants from funding organizations in the public, commercial, or non-profit sectors.

## Declaration of competing interest

The authors declare that they have no known competing financial interests or personal relationships that could have appeared to influence the work reported in this paper.
